# Fibroblast activation protein-based theranostics in pancreatic cancer

**DOI:** 10.3389/fonc.2022.969731

**Published:** 2022-10-03

**Authors:** Chien-shan Cheng, Pei-wen Yang, Yun Sun, Shao-li Song, Zhen Chen

**Affiliations:** ^1^ Department of Integrative Oncology, Shanghai Cancer Center, Fudan University, Shanghai, China; ^2^ Department of Oncology, Shanghai Medical College, Fudan University, Shanghai, China; ^3^ Department of Traditional Chinese Medicine, Shanghai Jiao Tong University School of Medicine Affiliated Ruijin Hospital, Shanghai, China; ^4^ Department of Research and Development, Shanghai Proton and Heavy Ion Center, Fudan University Cancer Hospital, Shanghai, China; ^5^ Nuclear Medicine Department, Shanghai Cancer Center, Fudan University, Shanghai, China

**Keywords:** pancreatic adenocarcinoma, fibroblast activation protein-α, cancer-associated fibroblasts, FAPI imaging, cancer theranostics

## Abstract

Fibroblast activation protein-α (FAP) is a type II transmembrane serine protease that has specific endopeptidase activity. Given its well-established selective expression in the activated stromal fibroblasts of epithelial cancers, although not in quiescent fibroblasts, FAP has received substantial research attention as a diagnostic marker and therapeutic target. Pancreatic cancer is characterized by an abundant fibrotic or desmoplastic stroma, leading to rapid progression, therapeutic resistance, and poor clinical outcomes. Numerous studies have revealed that the abundant expression of FAP in cancer cells, circulating tumor cells, stromal cells, and cancer-associated fibroblasts (CAFs) of pancreatic adenocarcinoma is implicated in diverse cancer-related signaling pathways, contributing to cancer progression, invasion, migration, metastasis, immunosuppression, and resistance to treatment. In this article, we aim to systematically review the recent advances in research on FAP in pancreatic adenocarcinoma, including its utility as a diagnostic marker, therapeutic potential, and correlation with prognosis. We also describe the functional role of FAP-overexpressing stromal cells, particulary CAFs, in tumor immuno- and metabolic microenvironments, and summarize the mechanisms underlying the contribution of FAP-overexpressing CAFs in pancreatic cancer progression and treatment resistance. Furthermore, we discuss whether targeting FAP-overexpressing CAFs could represent a potential therapeutic strategy and describe the development of FAP-targeted probes for diagnostic imaging. Finally, we assess the emerging basic and clinical studies regarding the bench-to-bedside translation of FAP in pancreatic cancer.

## 1 Introduction

**Figure 1:**
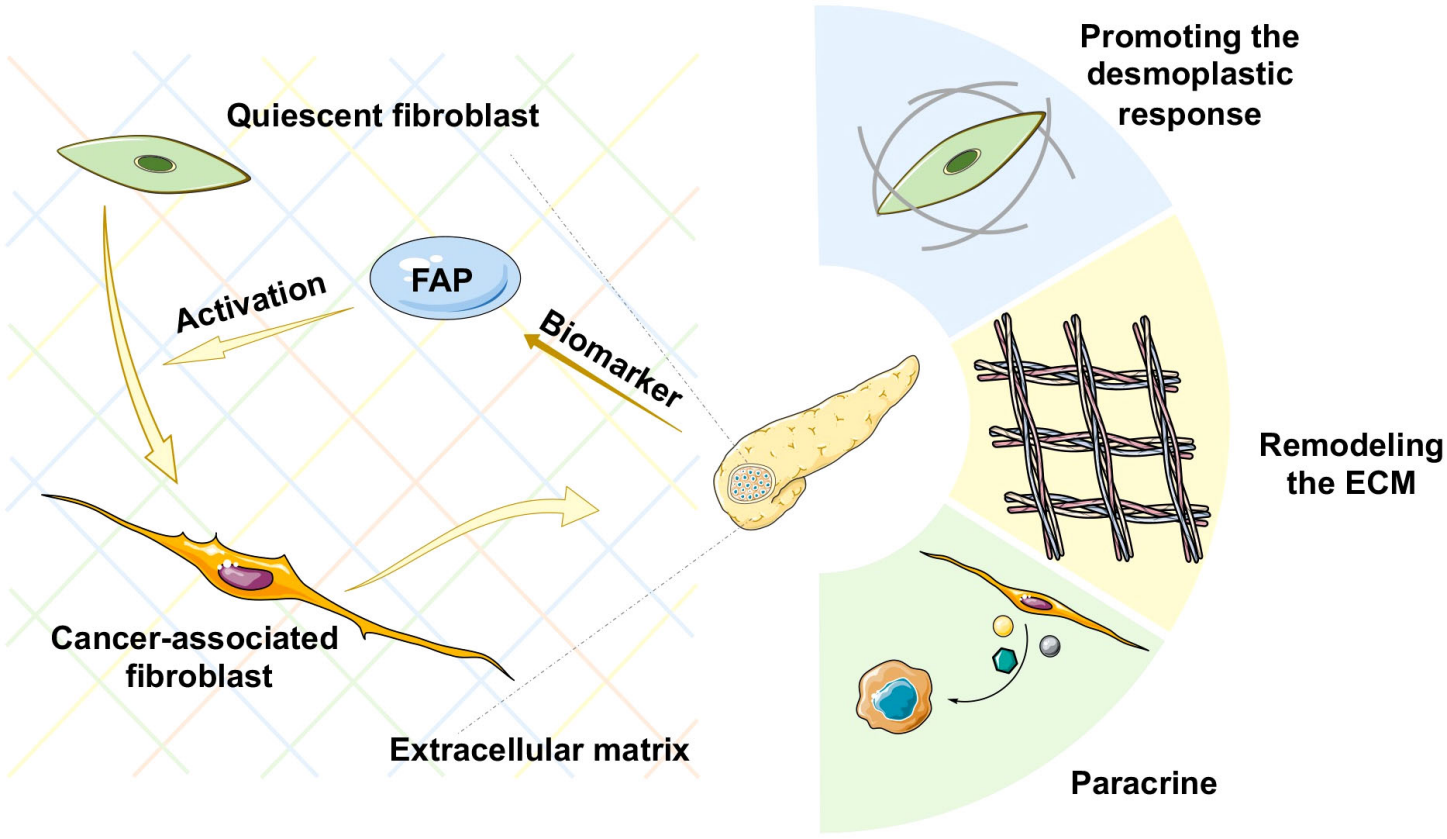
Schematic representation of various mechanisms of cancer-associated fibroblast (CAF) activation in pancreatic cancer. ECM, extracellular matrix.

Pancreatic adenocarcinoma (PAAD) is a highly aggressive and lethal cancer requiring novel diagnostic and therapeutic approaches. Characterized by an abundant fibrotic and extensive desmoplastic stromal response, cancer-associated fibroblasts (CAFs) are the most abundant component of the PAAD stroma. CAFs accumulate in pancreatic tumor tissue, wherein they enhance collagen family protein and fibronectin expression in a paracrine manner, thereby maintaining and remodeling the extracellular matrix structure. They also generate multiple factors, including exosomes, growth factors, immune- and metabolism-associated metabolites in an autocrine manner to communicate with cancer cells. Moreover, they contribute to promoting the desmoplastic response and mediate the early invasion, high recurrence rate, and treatment resistance of PAAD (1). Owing to abundant extracellular matrix deposition, vasculature, and fibroblasts, the pancreatic cancer stroma acts as a firm haven protecting cancer cells from different interventions, thereby contributing to treatment resistance. Thus, rather than playing the roles of a mere tumorigenic onlooker, the desmolastic stroma functions as an active participant in pancreatic cancer progression, invasion, migration, metastasis, immunosuppression, and resistance to treatment. Therefore, CAFs have become an attractive therapeutic target in the stroma of pancreatic cancers (**Figure 1**).

Fibroblast activation protein-α (FAP), named after its activating role, is a type II transmembrane serine protease with specific endopeptidase activity that is upregulated in different cancer types. Given its well-established selective expression in the activated stromal fibroblasts of epithelial cancers, although not in quiescent fibroblasts, FAP has received considerable research attention as a diagnostic marker and therapeutic target. FAP was first identified in 1986 by Wolfgang Rettig based on its role in activating fibroblasts (2). Initial research conducted on FAP focused on its role as a cell surface antigen. Subsequent studies, however, revealed that FAP could be shed from the cell membrane, thereby undergoing conversion to a soluble derivative referred to as circulating antiplasmin-cleaving enzyme (APCE) (3). In normal tissues and plasma, FAP is typically expressed at low levels and appears to serve as a redundant or non-essential protease in developmental processes. However, FAP was subsequently established to be involved in diverse pathological processes, including wound healing; inflammation, such as arthritis, atherosclerosis, and fibrosis; and epithelial cancers. The abundant expression of FAP in cancer and stromal cells, and particularly in CAFs, has been implicated in different cancer-related signaling pathways, thereby contributing to cancer progression, invasion, migration, metastasis, immunosuppression, and resistance to treatment. Consequently, FAP^+^ CAFs have emerged as important regulators and potential treatment targets.

The high levels of FAP expression in pancreatic cancer stroma have also been correlated with the poor prognosis of patients and desmoplasia (4, 5), and FAP has been identified as an independent predictor of pancreatic cancer survival (6, 7). The extensive fibrotic matrix of PAAD serves as a physical barrier that contributes to resistance to chemoradiotherapy or immunotherapy. Treatment paradigms targeting cancer cells are unable to overcome this obstacle, and consequently, considerable research effort has been devoted to characterizing the mechanisms underlying the treatment resistance of PAAD and identifying novel therapeutic strategies that can target the associated stroma and fibroblasts. In this review article, we summarize the promising theragnostic role of FAP in PAAD, with the aim of gaining further insights regarding the clinical implications.

## 2 Clinicopathological significance and prognostic value of FAPin PAAD

### 2.1 FAP expression and its clinical significance in PAAD

According to the National Cancer Institute Clinical Proteomic Tumor Analysis Consortium (CPTAC) dataset, FAP protein expression is significantly higher in primary PAAD tumor tissues than in normal tissues. Similarly, stromal FAP expression has been detected in approximately 98% of specimens in a sample of 48 patients with surgically resected PAAD (7). Consistently, the findings of a further immunohistochemical study involving 134 patients with PAAD revealed that FAP was expressed in both stromal fibroblasts (98/134, 73.1%) and cancer cells (102/134, 76.1%), with expression being significantly associated with patient age (*p* < 0.001), tumor size (*p* < 0.001), fibrotic foci (*p* = 0.003), and perineural invasion (*p* = 0.009) (4), thereby highlighting the importance of FAP in the clinicopathological characterization of PAAD.

In humans and some mammals, plasma contains small amounts of FAP, at concentrations of approximately 100 ng/mL or 0.6 nmol/L (8). FAP is also detectable in peripheral blood, the levels of which have been used for disease characterizations. For example, FAP has been used as a molecular marker in the identification of subpopulations of circulating tumor cell (9). Moreover, the measurement of FAP-specific substrates in body fluids has also been reported (10). However, as to whether the levels of FAP enzyme activity can serve as an informative diagnostic tool and in the assessment of disease progression in the clinical setting has yet to be sufficiently ascertained. Although the potential utility of FAP immunohistochemistry and plasma FAP as a biomarker in renal tumors for the differential diagnosis at an early stage have previously been reported (11), there have to date been no comparable studies with respect to pancreatic cancer.

### 2.2 FAP as a prognostic marker in PAAD

**Figure 2:**
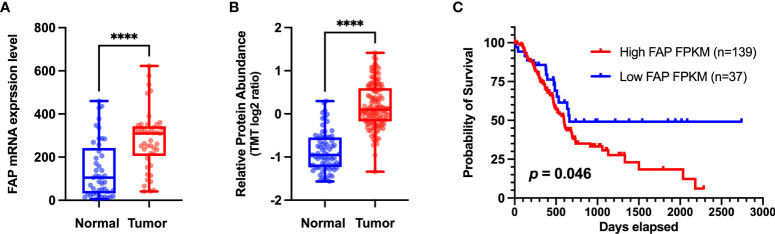
**(A)** Different levels of FAP mRNA expression in normal and tumor tissues. Transcriptome data of pancreatic adenocarcinoma (PAAD) and matched normal tissue were obtained from the Gene Expression Omnibus dataset (http://www.ncbi.nlm.nih.gov/geo) (GSE28735) for validation at the transcriptomic level. **(B)** Different levels of FAP protein expression in normal and tumor tissues. Proteome data were obtained from the CPTAC dataset (12) for validation at the protein level. **(C)** Overall survival analysis of two groups of patients with PAAD from The Cancer Genome Atlas (TCGA) database (https://portal.gdc.cancer.gov/). The Kaplan–Meier method was used to compare the survival of different FAP expression groups (the expression cutoff = 2.8), and the log-rank method was used for statistical analysis. GraphPad 9.0 software was used for data visualization. ****p < 0.0001.

The overexpression of FAP has also been implicated in the poor prognosis of PAAD patients (shown in **Figure 2**). Survival analyses have revealed that high FAP expression is associated with lower overall survival (12). A KPC mice model in which FAP had been deleted was characterized by delayed primary tumor onset and a more prolonged survival (13). Studies have also shown that higher CD8 expression and lower FAP expression in stromal cells can independently predict the prognosis in PAAD patients, and that blockade of FAP may improve the prognosis of patients with PAAD. It may increase CD8 cell levels (6). Furthermore, a comprehensive transcriptome analysis (14) identified two four-hub gene modules (including FAP) as specific predictive features for PAAD diagnosis and prognosis, thereby providing a new perspective with respect to clinical trials. Moreover, by applying weighted gene co-expression network analysis, Wang et al. (15) established that FAP is involved in a core module associated with PAAD type. Collectively, the findings of these studies highlight the potential utility of FAP expression patterns as a prognostic marker and an immune checkpoint target in PAAD.

## 3 The role of FAP in the PAAD tumor microenvironment

**Figure 3:**
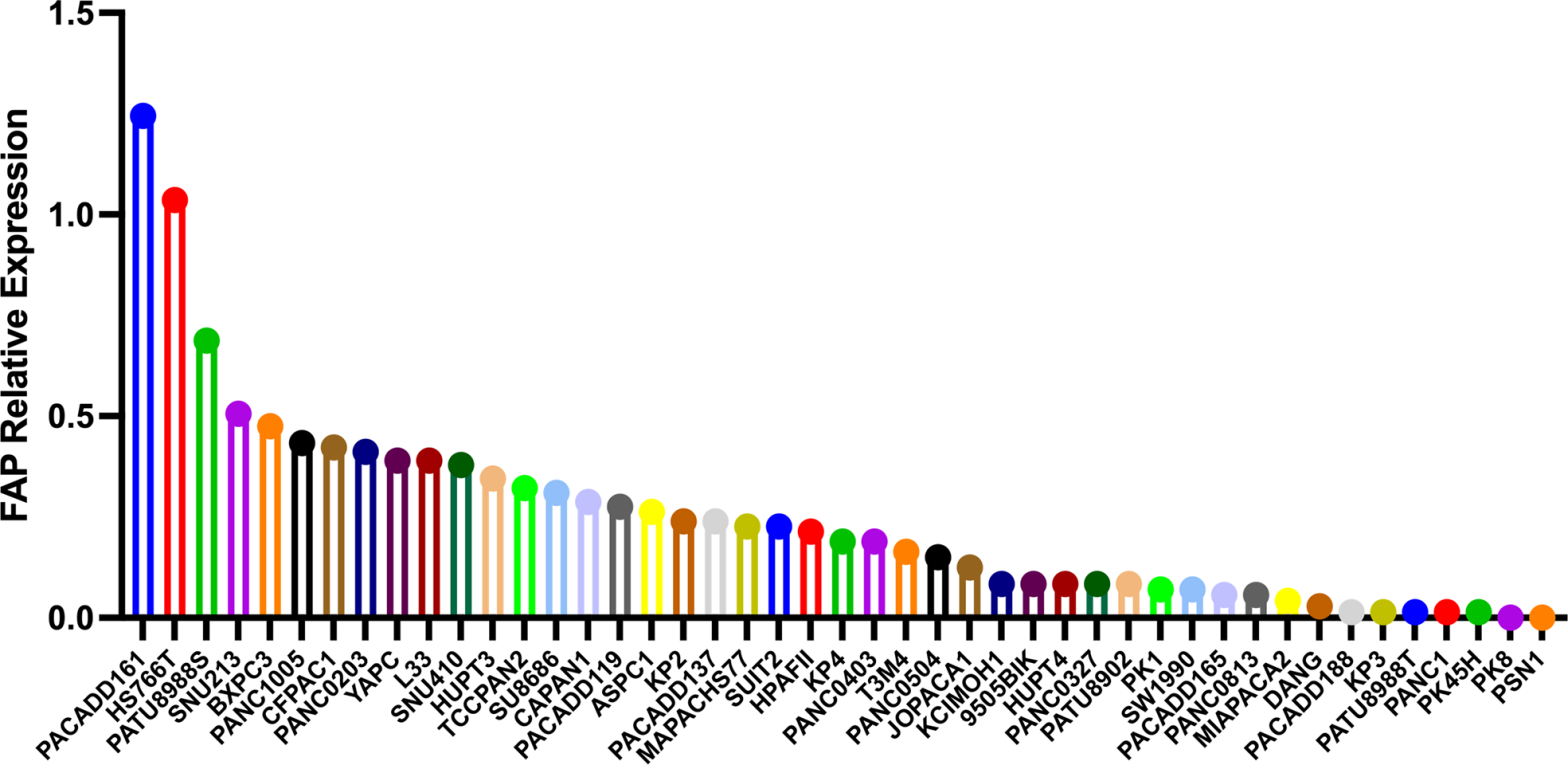
The different mRNA levels of FAP protein expression in pancreatic cancer cell lines using data obtained from the Motivations for the Cancer Cell Line Encyclopedia database (https://portals.broadinstitute.org/ccle).

**Figure 4:**
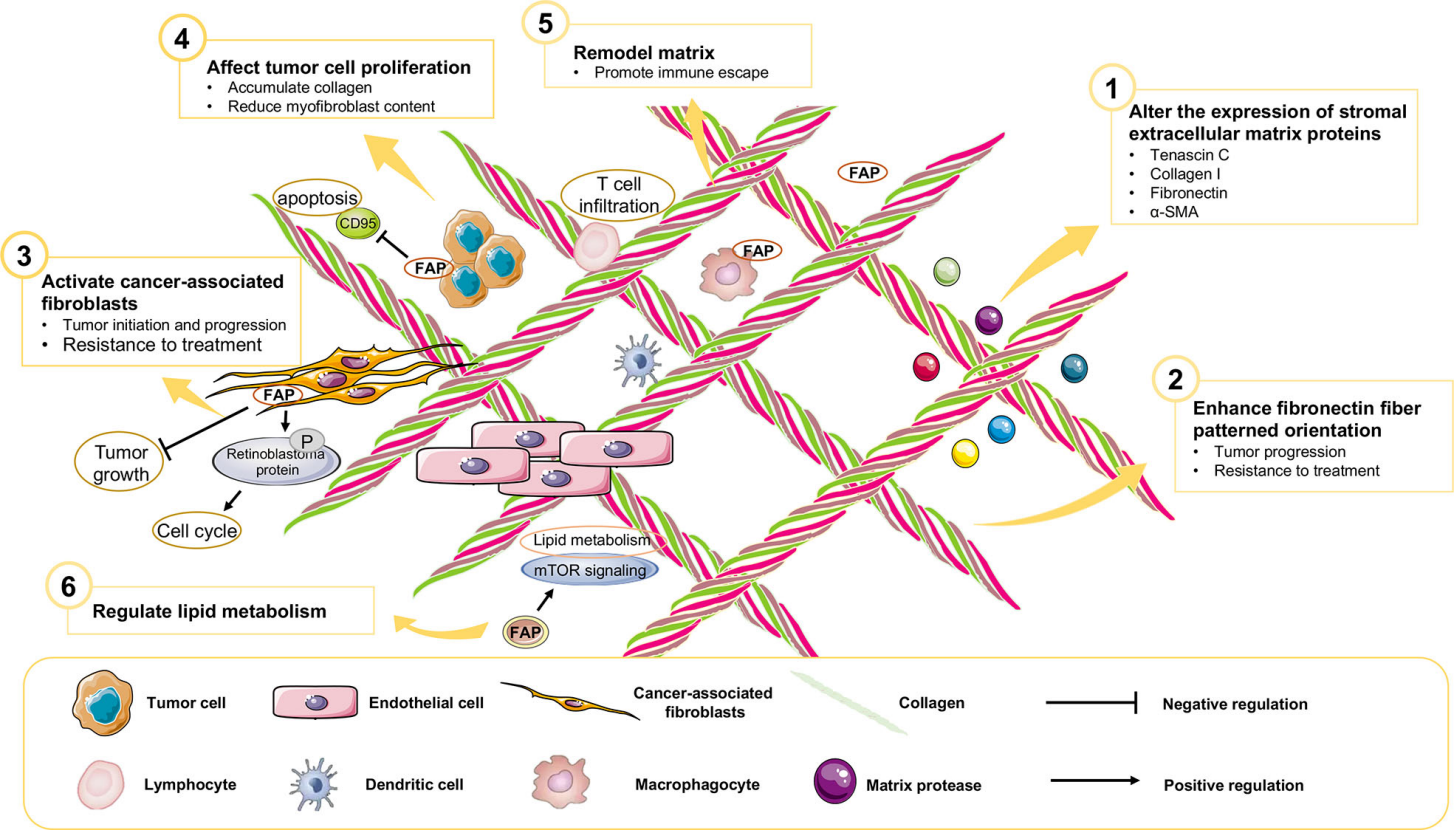
The role of FAP in the PAAD tumor microenvironment.

**Figure 5:**
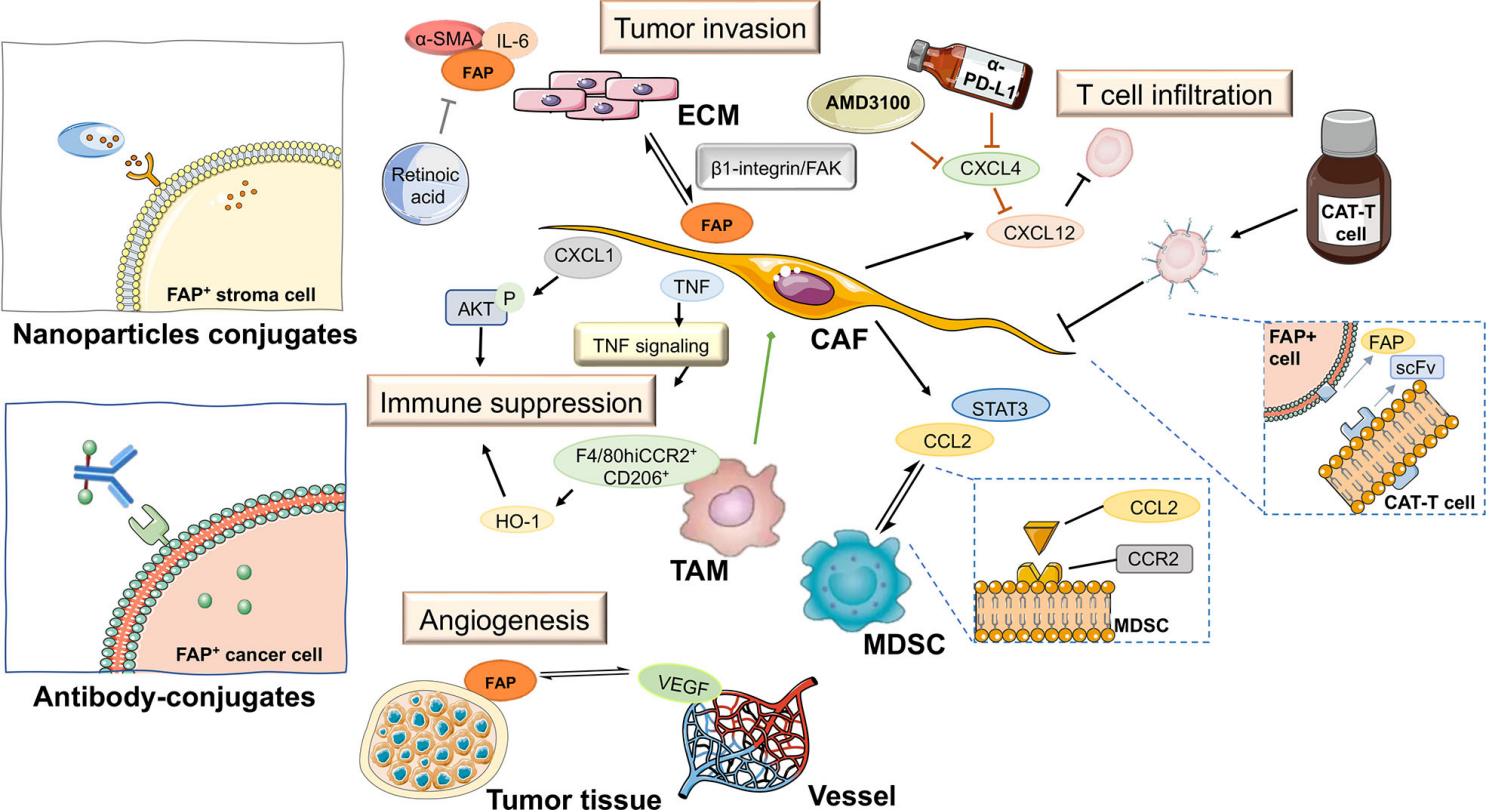
FAP-targeted therapies and their expected impact on pancreatic tumor tissue. ECM, extracellular matrix; TAM, tumor-associated macrophage; CAT-T cell, chimeric antigen receptor T cell; MDSC, myeloid-derived suppressor cell; CAF, cancer-associated fibroblast; scFv, fragments of single-chain antibody.

Previous diagnostic and therapeutic paradigms have tended to focus almost exclusively on tumor cells, and have failed to make any significant breakthroughs in PAAD therapy during the past decade. In this context, it has been established that FAP is expressed predominantly in stromal cells, whereas only very low levels of expression are detected in cancer cells in resected pancreatic ductal adenocarcinoma (PDAC) tissues (7). At the cellular level, pancreatic cancer cell lines have been shown to express FAP protein to a greater or lesser extent (4), and mRNA at different levels (**Figure 3**). Furthermore, the expression of FAP has been shown to alter the expression of stromal extracellular matrix proteins, including tenascin C, collagen I, fibronectin, and α-SMA, and also to enhance fibronectin fiber patterned orientation, thereby resulting in the rapid disease progression and increasing resistance to treatment (16). PAAD cells communicate with stromal cells, recruit fibroblasts, and activate these to yield cancer-associated fibroblasts. The finding of numerous studies have established the link between stromal-expressed FAP, particularly FAP^+^ CAFs, and pancreatic cancer, thereby indicating the potential ultility of FAP as a target in PAAD treatment (**Figure 4**). Moreover, it has been found that FAP^+^ stroma promotes cancer immune escape (17). In addition, findings relating to the enzymatic role, expressional patterns, and stroma modulation role of FAP in PAAD have provided insights for further examination of its pro-tumorigenic role and provided evidence to indicate that FAP could serve as both a novel marker in PAAD diagnosis and as therapeutic target (**Figure 5**).

### 3.1 The enzymatic role of FAP

FAP is a member of the dipeptidyl peptidase (DPP) 4 family of proteins that hydrolyzes a prolyl bond at sites two amino acids from the N-terminus of a protein. The cyclic character of proline residue makes targeting the specific catalytic ability of FAP attractive. However, the finding that mice in which FAP is not expressed show no an aberrant phenotype would tend to indicate that under physiological conditions, FAP is a redundant or non-essential protease (18).Moreover, in preclinical or clinical research, it has been demonstrated that inhibitors of FAP fail to have significant anti-cancer effect in preclinical or clinical research (19–21). These observations could be ascribed to one of several possible factors. Firstly, FAP could act indirectly on tumor cells, as has been indicated by the finding that the depletion of FAP indirectly inhibits tumor cell proliferation *via* an enhancement of collagen accumulation and the impediment of myofibroblast content (19). Mechanistically, it has been found that the regulation of FAP-1 enzymatic activity negatively regulates CD95 (Fas, APO-1)-mediated apoptosis in pancreatic cancer cells (22). Additionally, as a known collagen-degrading enzyme, FAP mediates matrix remodeling and in part regulates lipid metabolism *via* mTOR signaling (23). Secondly, the development of talabostat as an anti-cancer agent has focused mainly on its immune-mediated activity, and to date, there have been no studies that have specifically sought to evaluate the efficacy of talabostat in FAP-expressing tumors, or its use in combination with other targeted therapies. Collectively, these findings provide evidence to indicate a potential regulatory role of FAP under pathological conditions, and that this FAP-mediated regulation of lipid metabolism and remodeling of the extracellular matrix (ECM) could serve as a potential novel therapeutic target.

### 3.2 FAP^+^ cells within the PAAD tumor microenvironment

Despite efforts designed to suppress the expression and enzymatic activity of FAP as anti-cancer treatments, studies, such as those involving the development of a murine anti-FAP mAb F19 and its humanized version unconjugated sibrotuzumab (BIBH 1) (24–26), have failed to demonstrate promising clinical efficacy. On the basis of the studies conducted to date, there is no evidence to indicate that direct targeting of FAP would be either safe or effective. Consequently, given the overexpression of FAP in a wide variety of stromal cells during tumorigenesis, this would tend to identify the stroma as a target for anti-cancer drugs. Indeed, it has been found that depletion of FAP^+^ cells has the effect of delaying subcutaneous tumor growth and contributes to the development of a more immunogenic environment, thereby indicating that FAP-expressing stromal cells could play important roles as immunomodulators (27).

Malignant cells drive the generation of desmoplastic and immunosuppressive tumor microenvironments, and emerging evidence indicates that FAP is aberrantly expressed in CAFs, chimeric antigen receptor T (CAR-T) cells, and a subset of M2 macrophages. Furthermore, FAP-expressing cells have also been associated with the degradation of extracellular matrix proteins and glycosaminoglycans, as well as a reduction in tumor vessel density, which jointly contributes to the continual remodeling of the tumor microenvironment during the progression of PAAD. Furthermore, the emerging development of nanoparticle conjugates and antibody-conjugates is offering a potentially wide range of therapeutic options from the perspective of targeting FAP in cancer.

#### 3.2.1 FAP^+^ CAFs associated with immunosuppressive tumor stroma

CAFs are the main component of the PAAD stroma, thereby indicating that targeting CAFs could represent a viable therapeutic strategy. CAFs play a pleiotropic role in PAAD initiation and progression, and in line with expectations, it has been demonstrated that depletion of CAFs can accelerate tumor progression (28, 29). In this context, attention has focused on targeting precise subpopulations of CAFs that are known to be oncogenic and pro-progression, particularly FAP-positive CAFs. These FAP-expressing CAFs have been proposed to accelerate proliferation, invasion, and therapeutic resistance in ovarian cancer cells (30), and have also been suggested to reprogram tumor inflammatory environments in human intrahepatic cholangiocarcinoma *via* FAP-STAT3-CCL2 signaling (31). However, in-depth research on PAAD cells is still lacking. By promoting retinoblastoma protein phosphorylation, FAP^+^ CAFs have been suggested to alter the cell cycle of co-cultured PAAD cells (7). Furthermore, the depleting of FAP-expressing cells (including all FAP^+^ CAFs), has been found to delay tumor growth in a subcutaneous model of PAAD (27).

It is well established that PAAD is highly resistant to immunotherapy, and it has been demonstrated that FAP-positive stromal cells (including almost all the CAFs) mediated local immunosuppression and immune escape, thereby compromising the efficacy of immunotherapy, and in particular, immune checkpoint blockade treatment (27, 32). Studies have indicated that the production of chemokine (C-X-C motif) ligand 12 (CXCL12) by FAP^+^ CAFs within the cancer cell-rich region of a tumor impedes T cell infiltration leading to immunosuppression (32). To counteract this suppressive effect, a synergized treatment approach has been proposed, which entails inhibition of the CXCL12 receptor by the suppression of chemokine (C-X-C motif) receptor 4 (CXCR4) using α-PD-L1, a checkpoint antagonist. This was demonstrated to promote T-cell accumulation and contribute to tumor regression (32). Recently, a CXCR4 small-molecule inhibitor, AMD3100 (plerixafor), was assessed in a phase I clinical trial (NCT02179970) that aimed to evaluate its safety in PAAD patients. The findings of the comparative transcriptional analysis revealed an unusually rapid enhancement of intra-tumoral B and T cell responses within 7 days of treatment, thereby indicating the possible induction of an integrated anti-cancer immune response. Given these promising findings, further clinical trials are warranted that evaluate responses in a larger number of patients who have undergone repeated cycles of combination therapy with CXCR4 inhibitor and immune checkpoint antagonist, along with additional studies examining tolerability, pharmacokinetics, and pharmacodynamics to further assess the therapeutic activity.

However, the fact that several well-established biomarkers of CAFs are also typically expressed on the surface of other cell types, raises the likelihood that CAF-targeted therapy might have certain unwanted side effects. As an alternative strategy for the treatment of PAAD, researchers are examining the effects of blocking the interactions between CAFs and their neighboring cells. Determining the mechanisms underlying the accumulation of CAFs in cancer and developing methods that can be used to identify different CAF subsets in the tumor microenvironment will contribute to establishing therapeutic strategies that could convert the tumor-promoting microenvironment into a tumor-suppressing microenvironment. Considerable evidence has accumulated to indicate that FAP plays an important role in this alteration, and thus further research on the synergetic roles of FAP^+^ CAFs in PAAD immunotherapy is warranted.

#### 3.2.2 Crosstalk between FAP^+^ CAFs and MDSCs and TAMs

The infiltration of immature myeloid cells, including the myeloid-derived suppressor cells (MDSCs) and macrophages, is one of the well-defined hallmarks of cancer (33). In this regard, Yang et al. (31) have demonstrated that FAP^+^ CAFs can enhance the recruitment of MDSCs *via* STAT3-CCL2 signaling and thereby promote tumor progression in a murine liver tumor model. FAP^+^ CAFs are characterized by a distinct inflammatory phenotype of STAT3 activation and pro-inflammatory cytokine CCL2 upregulation. As the primary source of CCL2, FAP^+^ CAFs undergo crosstalk with circulating MDSCs expressing the CCL2 receptor (CCR2), thereby forming a CCL2–CCR2 axis that plays a role in mediating tumor promotion. In line with expectations, it has been observed that the tumor progression promoting and MDSC recruiting effects of FAP^+^ CAFs are abolished in Ccr2-deficient mice. However, current evidence indicates that the activity of tumoral FAP^+^ stromal cells contributes to immune suppression, the function of FAP^+^ CAFs in the recruitment of MDSCs in PAAD remains largely undetermined, and accordingly warrants further investigations.

Recently, a direct mechanism has been proposed for the generation of CAFs, which indicates that tumor-associated macrophage (TAMs) expressing CAF markers could be a potential source of CAFs (34, 35). The findings of research have indicated that these FAP-positive cells comprise two subpopulations, namely, a CD45^−^ mesenchymal subset and a CD45^+^ hematopoietic subset (36). In this regard, a previous study on immunogenic Lewis lung carcinoma cells identified FAP^+^CD45^+^ cells as a subset of F4/80^hi^CCR2^+^CD206^+^ M2 macrophages and the main tumoral source of the immune inhibitory enzyme heme oxygenase-1 (HO-1). The depletion of FAP^+^CD45^+^ cells or administration of the HO-1 inhibitor Sn mesoporphyrin has been shown to promote the immune-dependent arrest of subcutaneous tumor growth (36). Moreover, FAP^+^/F4/80^+^/HO-1^+^ stromal cells have been identified in a PAAD subcutaneous tumor model. Sn mesoporphyrin has been established to induce an immune response to a currently unidentified antigen(s) and inhibits tumor growth in a tumor-bearing mouse model of PAAD (37). In PAAD, TAMs in the tumor stroma also secretes FAP, shifting their phenotype to an immunosuppressive M2 macrophage type, suppressing the adaptive immune response, and promoting a positive feedback loop in PAAD progression (37).

#### 3.2.3 T cells and tumor-infiltrating lymphocytes

PAAD is histologically characterized by a large stroma volume, and previous studies have consistently reported the suppression of intra-tumoral effector T-cells by the FAP-expressing stromal cells among different cancer types (27, 32, 38). In PAAD, FAP^+^ CAF-abundant areas have been shown to be characterized by limited CD8^+^ lymphocytes infiltration, thereby indicating the contribution to the spatial exclusion of intra-tumoral CD8^+^ T cells (39), along with Tregs and neutrophil accumulation and cancer-associated pathways modulation (40). Given that heterogeneous and differential FAP^+^ CAFs in PAAD tumors may be involved to varying degrees in stromal variation, immunosuppression, and differential responses to potential immunotherapy, further experimental studies are necessary to clarify the potential causal inference.

#### 3.2.4 The extracellular matrix and tumor vascularity

In addition to cellular constituents, important components of the tumor microenvironment include the ECM, blood vessels, and cytokines (41). FAP activity has been found to direct stromal ECM organization *via* β1-integrin-dependent cell matrix interactions, in which suppression of FAP activity limits β1-integrin/FAK-mediated invasive capacity in PAAD cells (16). Moreover, the findings of a further study have indicated that retinoic acid, a vitamin A derivative, plays a role in transforming FAP-activated CAFs into static fibroblasts *via* the down-regulated expression of α-SMA, FAP, and IL-6 and the inhibition of the ECM production (42). These findings provide important insights regarding the selective targeting of the main source of ECM proteins and highlight the potential utility of FAP-mediated disruption of the ECM as a novel therapeutic approach for PAAD.

Levels of vascular endothelial growth factor (VEGF), a promotor of angiogenesis and biomarker of vessel density, are found to be raised in PDAC tissues and are linked to liver metastases and poor prognosis. It has previously been demonstrated that in the cancerous tissues of PAAD patients, the expression of FAP is positively correlated with VEGF (41). This significant positive correlation between FAP and VEGF indicates that the two factors conjunctly influence PAAD survival, and that contributing to the complexity of these interactions are pericytes, inflammatory cells, and fibroblastic cells, which together with PAAD cells, shape the tumor vasculature.

Cytokines and chemokines, including tumor necrosis factor (TNF), and CXCL1, secreted by FAP^+^ CAFs contribute to the further suppression of adaptive immunity in PAAD (38, 43) and are currently attracting considerable attention among researchers. Moreover, with an increasing number of studies focusing on TNF, its roles in fibroblasts within the tumor microenvironment have gradually become more precisely elucidated, and it has accordingly been established that its functions are not confined to merely driving fibroblast activation. However, the tumor-promoting and immunosuppressive activity of FAP^+^ fibroblasts have been demonstrated to suppress TNF signaling (27, 44, 45), thereby contributing to cancer–stroma–cancer interaction loop that promotes tumor progression (46). For example, the pancreatic stellate cells (PSCs), the FAP^+^ resident cells of the pancreas, have been implicated in cancer-related fibrosis, induction of PAAD migration, and invasion *via* the activation of CXCL1-mediated AKT phosphorylation. Furthermore, the secretion of transforming growth factor-β1 (TGF-β1) PAAD cells has been observed to induce the expression of FAP in hitherto quiescent PSCs, thereby promoting a further releasing CXCL1, promoting tyrosine kinase receptors phosphorylation, and forming a positive feed-forward loop promoting PAAD progression (47). Collectively, the multifarious aspects of the tumor microenvironment provide a pool of secreted cytokines that stimulates or suppresses different components of this microenvironment, thereby contributing to the progression of PAAD.

## 4 Immunotherapeutic targeting of FAP^+^ cells

As previously mentioned, the FAP^+^ stroma and CAFs play pivotal roles in mediating the immunosuppressive characteristics of PAAD (32) and are responsible for the ineffective activity of known T-cell checkpoint antagonists. Cytotoxic T-lymphocyte-associated protein 4 (CTLA-4) and programmed cell death protein 1 (PD-1) or its ligand PD-L1 function as important checkpoint receptors on T cells, and accordingly present ideal targets for inhibition by antagonistic antibodies. However, given the ineffectual nature of immunotherapy and therapeutic approaches. In this regard, targeting tumoral FAP^+^ stromal cells is seen as a novel alternative therapeutic option. Emerging evidence indicates that the malignant behavior of PAAD, such as resistance to treatment, immune escape, and metastasis, is associated with the complex interaction between stromal and tumor cells. However, the current therapeutic paradigm focuses on tumor-stroma interactions in PAAD rather than the expression of FAP protein. It is believed that gaining a better understanding of the role of this protein in the FAP-mediated interaction between stroma and tumor cells could contribute to optimizing the FAP-inhibition strategy.

### 4.1 The FAP^+^ CAR-T

Chimeric antigen receptor T cells (CAT-T cells) are genetically engineered T cells that are becoming increasingly widely used as components in novel types of anti-cancer immunotherapy. T cells contain fragments of a single-chain antibody that can recognize predefined surface antigens, thus enabling T cells to effectively identify and target cancer cells, and thereby affording a considerable range of opportunities to modify and improve CAR T cell therapy. Traditionally, predefined surface antigens on cancer cells have been the primary targets in anti-cancer therapy. However, the findings of recent studies have identified a number of antigens, which, although expressed at very low levels on normal cells, are highly expressed on the surface of tumor stromal cells, thereby indicating their potential value as novel targets for CAR T cell therapy that can minimize on-target/off-tumor toxicity. This has thus stimulated an increasing interest in the development of CAR tools with FAP specificity for T cell modification.

Studies using mouse models have shown that FAP-CAR T cells can exhaust FAP-expressing stromal cells and inhibit tumor growth by producing immunostimulatory cytokines, promoting tumor cytolysis (48), and enhancing the anti-tumor response of endogenous CD8^+^ T cells (49). Moreover, synchronously targeting both malignant cells and FAP^+^ stroma has been established to produce a robust anti-tumor effect. However, the findings of a further study have indicated that the presence of highly reactive FAP-specific CAR-modified T cells can promote severe cachexia and dose-limiting bone toxicity without significantly affecting tumor suppression among different syngeneic tumor implantation models in mice. These observations tend to indicate that FAP-expressing stroma derived from different sources might contribute to contrasting outcomes. Hence, this emphasizes the necessity to take into consideration the location of FAP expression in stromal cells during PAAD initiation and progression and the requirement for a more precise biomarker of FAP+ CAFs. These results warrant further assessment for optimizing FAP-specific CAR in preclinical and clinical trials of human PAAD.

The findings of studies that have used murine pancreatic cancer models have tended to indicate that the adoptive transfer of FAP-CAR T can also inhibit tumor growth in an immune-independent manner (50–52). Furthermore, the adoptive transfer of FAP-targeted CAT-T cells has been demonstrated to reduce tumor vascularity, ECM proteins, and glycosaminoglycans (48, 53, 54). Moreover, FAP-specific CAR T transfer has been found to selectively recognizes and depletes the FAP^+^ subsets of cancer stem cells, which play a vital role in maintaining the tumor stroma and eliminating FAP^+^ CAFs. Consequently, the adoptive transfer of FAP-CAR T has provided insights that will contribute to the further development of FAP^+^-specific stromal cell-targeted therapies for treating PAAD by targeting FAP^+^ cells in the tumor microenvironment.

### 4.2 Pd-1/Pd-Li

Investigations that have focused on the depletion of FAP^+^ cells in the PAAD tumor microenvironment have revealed an anti-tumor effect associated with α-CTLA-4 and α-PD-L1 treatment, thereby providing further evidence in support of the immune-suppressive role of FAP^+^ stromal cells in the poor responsiveness to T-cell checkpoints antagonists. As previously mentioned, CXCL12 produced by FAP^+^ CAFs may direct tumor immune evasion in a model of human PAAD and synergized treatment of CXCL12 receptor inhibition with α-PD-L1 (32), thereby remodeling the immunosuppressive microenvironment. In this context, Ji et al. (55) designed a cleavable amphiphilic peptide (CAP) marker to be specifically responsive to FAP stroma, in which, at the site of FAP^+^ cell abundance, the CAP was cleaved, and nanoparticles rapidly disassembled and unloaded the drug into a solid tumor. In addition, an amphiphilic bifunctional PD-1/PD-L1 peptide antagonist has been developed to deliver doxorubicin and R848 after being cleaved by FAP, which in combination with PD-1 blockade therapy was observed to trigger a stronger immune response on activating immunogenic cell death and reprogramming tumor-associated macrophages (56). Furthermore, it is worth noting that stromal factors such as TGF-β and FAP, which are commonly characterized by altered expression in PAAD, were found to be associated with resistance to neoadjuvant atezolizumab therapy (targeting PD-1, PD-L1) in operable urothelial carcinoma in the ABACUS trial (NCT02662309) (57). The findings of these studies thus indicate the utility of FAP as a clinical biomarker in T-cell checkpoint antagonist treatments, and consequently, have potential implications for comparable treatment in PAAD warrants further studies.

Currently, several clinical trials targeting FAP, especially RO6874281, are ongoing across cancers. An immunocytokine RO6874281 is composed of an interleukin-2 variant (IL-2v) targeting FAP and pembrolizumab (anti-PD-1), leading to the blockade of T cells migration. RO6874281 is applied to immunotherapy of several cancers, including renal cell carcinoma (NCT03063762), metastatic melanoma (NCT03875079), solid tumor, breast cancer, and cancer of head and neck (NCT02627274), and metastatic PAAD(NCT03193190). These trials are active or recruiting, without results yet, but their preclinical studies are promising. Therefore, it is valuable to keep a watchful eye on the results.

### 4.3 Novel nanoparticle conjugates and antibody-conjugates

As a protease, FAP has also been exploited in drug innovation studies, and several methods have been adopted that target FAP-expressing cells based on FAP enzyme activity. For example, nanomaterial‐based drug delivery systems are a common platform used to deliver drugs to tumor sites. In this regard, the stroma can act as a barrier that impedes the passage of drugs targeting tumor sites. However, the aberrant expression of FAP provides a strategy for overcoming this impediment, namely, the development of smart nanomaterials that respond to FAP-positive CAFs. Nanomaterial‐based drug delivery systems have shown considerable opportunity to optimize the drug specificity, biocompatibility, and pharmacokinetic features of these materials.

Sum et al. (58) have assessed a bispecific FAP-CD40 antibody that triggers a potent FAP-dependent stimulation of CD40, thereby enhancing tumor-specific T-cell priming and inducing tumor growth inhibition *in vivo*. By specifically inducing CD40 stimulation in the presence of FAP, this novel bispecific antibody was demonstrated to overcome the systemic toxicity associated with the CD40 agonist, thereby providing a promising approach for cancer immunotherapy. A further antibody-conjugate targeting FAP, FAP5-DM1, has also been shown to have an inhibitory effect on tumor progression, inducing complete regression in a PAAD xenograft model with good efficacy and tolerability (59). These studies have accordingly demonstrated the potential utility of combined targeting antibody conjugates as novel drug candidates for PAAD and stimulated further clinical translational studies toward clinical applications.

Although immune checkpoint blockade has shown promising results in the treatment of various cancers, the extensive fibrotic matrix and immunosuppressive effect of the PAAD tumor microenvironment have tended to hinder its wider application. In this respect, Yu et al. have proposed the use of novel thermo- and fibrotic matrix-sensitive liposomes encapsulating small-sized albumin nanoparticles loaded with an immune checkpoint inhibitor (BMS-202). Exposure to FAP and near-infrared laser radiation, with a mild elevation in localized temperature, induces the release of this agent at localized sites, thereby promoting the recovery of T lymphocyte activities within the immunosuppressive tumor microenvironment (60). By enhancing the accumulation of immune checkpoint inhibitors at the tumor site, these novel nanoparticles can promote the localized secretion of cytokines such as TNF-α and INF-γ, and potentiates the anti-tumor immune response in PAAD.

### 4.4 Safety concerns

Despite significant advances in research on FAP as an anti-cancer target candidate with broad clinical application prospects in conjunction with checkpoint blockade immunotherapy of solid tumors, the potentiality of lethal adverse effects should be taken into consideration when targeting FAP for immunotherapeutic purposes. The findings of previous studies have indicated that targeting FAP triggers the recognition of multipotent bone marrow stromal cells, whereas cachexic mouse and human pluripotent bone marrow mesenchymal stem cells (BMSCs) are recognized by FAP-reactive T cells (61). Fatal ototoxicity and cachexia observed following cell-based immunotherapy against FAP accordingly cautions against its use as a generic target. Furthermore, as FAP expression by pluripotent BMSCs may point to the cellular origin of tumor stromal fibroblasts and at least in part result from the universal recognition of FAP-reactive T cells on multipotent BMSCs, lethal bone marrow hypocellularity and necrosis, and cachexia have been observed subsequently to FAP-targeting immunotherapy (61). Additionally, long non-coding RNAs (lncRNAs), which are expressed in cell-type and disease-specific manners, have been demonstrated to contribute to the development and activation of CAFs (62, 63). In turn, these activated CAFs can promote tumor development by affecting the gene expression and secretory properties of cells, altering the tumor microenvironment, and enhancing malignant biological processes in cancer cells *via* lncRNAs. These observations provide evidence to indicate that FAP^+^CAFs-specific lncRNAs could potentially be targeted for PAAD therapy without affecting the FAP expression of normal cells. In addition to these plausible obstacles, other challenges remain and should be carefully assessed to avert the potential risks of systemic toxicity when targeting FAP. Accordingly, prior to translational research evaluating the utility of FAP-related therapy, a comprehensive assessment of the safety and toxicity of these treatments using different animal models is of the utmost necessity.

## 5 Targeting FAP^+^ CAF using diagnostic imaging and radioligand therapy probes

In preclinical and clinical trials, FAP-targeting molecular imaging radiotracers have shown promising results with respect to tumor diagnosis. At present, the clinical assessment of FAP-targeting radiotracers is primarily performed in small cohorts of patients in single-center studies. However, small sample populations and certain disparities among studies have led to difficulties in drawing definitive conclusions. Given the widely reported potential for oncological diagnosis, application of the [^68^Ga]Ga-FAPI-04 probe, for example, has been reported in clinical trials (NCT04554719 and NCT04605939) with the uptake of [^68^Ga]Ga-FAPI-04 in benign pancreatic lesions being demonstrated (64). Nevertheless, in low [^18^F]F-FDG-avid tumors, such as in PAAD, [^68^Ga]Ga-FAPI PET/CT shows high sensitivity in detecting primary pancreatic tumors, involved lymph nodes, and metastases, and is superior in terms of TNM staging (65), thereby indicating the potential utility of [^68^Ga]Ga-FAPI-PET/CT as an effective imaging tool. Consequently, upon diagnosis, it is essential to undertake a comprehensive pathological assessment.

Furthermore, when applied in combination with magnetic resonance imaging (MRI), the [^68^Ga]Ga-FAPI-04 PET/MR probe may enhance diagnostic sensitivity and prevent the misdiagnosis of certain pancreatic lesions. Although compared with the current gold standard contrast-enhanced CT, FAPI-PET/CT appears to be a superior imaging modality for pancreatic cancer (66), prospective trials with larger patient populations are needed to evaluate whether [^68^Ga]Ga-FAPI PET/CT can elicit treatment modification in PAAD when compared with other imaging methodologies. Similarly, a preliminary study has reported the diagnostic potential of ^18^F-FAPI-74 in FAP-expressing PAAD xenografts with higher tumoral uptake than [^68^Ga]Ga-FAPI-04, thereby highlighting the advantageous properties of ^18^F, notably the higher rate of detection and wide availability [https://jnm.snmjournals.org/content/62/supplement_1/1492/tab-article-info].

In pancreatic cancer, FAP-targeted radiotracers have been proposed as a diagnostic and therapeutic tool. For example, [^64^Cu]FAPI-04 and [^225^Ac]FAPI-04 have shown utility in theranostics for the treatment of FAP-expressing PAAD (67), whereas application of the albumin binder-conjugated FAPI radiotracers [^177^Lu]TEFAPI-06 and [^177^Lu]TEFAPI-07 has been found to promote substantial growth inhibition in patient-derived PAAD xenografts with negligible side effects (68). Other radiotracers, such as [177Lu]FAPI-46 and [225Ac]FAPI-4, have both demonstrated rapid renal clearance, relatively high intra-tumoral accumulation, and tumor-suppressive effects in PAAD xenografts, with a slight reduction in body weight (69). Furthermore, FAP-targeted radioligand therapy with ^90^Y-FAPI-46 has also been assessed in three patients with advanced PAAD (70). The administration of low radiation doses to at-risk organs suggests the feasibility of repeat cycles of ^90^Y-FAPI-46 with well-tolerated treatment and a low rate of attributable adverse events. However, although evidence of tumor response was observed following ^90^Y-FAPI-46 treatment, further studies are warranted to determine efficacy and the toxicity profile in a larger cohort (71).

Given that the FAP is generally expressed at low levels on non-malignant cells, numerous studies have focused on targeting FAP^+^ CAF to achieve precise imaging of solid tumors and assess potential treatments (72). Since the development of [^89^Zr]Zr-B12 IgG as a selective imaging probe for FAP-expressing tumors, a series of compounds with the general structure of EB-FAPI-Bn have been synthesized based on FAP inhibitor (FAPI) variants (73), including 99mTc-labeled FAPI tracers for SPECT imaging and 188Re therapy (74). However, the rapid clearance of these molecules and their inadequate tumor retention have hindered them from further clinical translation into cancer therapeutics.

## 6 Conclusion

Examination of the complex and highly heterogeneous characteristics of tumors and the tumor microenvironment may lead to the development of novel treatments that can contribute to reducing suffering and enhance the overall prognosis of PAAD patients. Studies have shown that the desmoplastic response in PAAD is not only a “bystander” but also a source of dynamic cellular and non-cytokinetic factors that promote tumor progression, immunosuppression, and metastasis. To date, the findings of numerous studies have confirmed the potential value of FAP expression patterns as prognostic markers and immune checkpoint targets in PAAD. They have also unraveled the role of this enzyme in crosstalk with other cellular components of the tumor microenvironment, as well as non-cellular components, such as cytokines and chemokines, and have thereby provided insights on angiogenesis, matrix remodeling, and immunosuppression.

As an oncogenic subset of CAFs, FAP^+^ CAFs mediate local immunosuppression and immune escape. FAP^+^ CAFs have emerged as a therapeutic paradigm for new PAAD drug candidates with the development of novel nanoparticle conjugates and antibody-conjugates, which will contribute to overcoming matrix barriers to drug delivery, thereby facilitating the rapid accumulation of immune checkpoint inhibitors at tumor sites. Moreover, FAP-specific modification of CAR T cells can contribute to the depletion of FAP-expressing stromal cells and inhibit tumor growth by promoting the production of immunostimulatory cytokines, inducing tumor cell lysis, enhancing the anti-tumor response of endogenous CD8^+^ T cells, and favoring the anti-tumor effects of α-CTLA-4 and α-PD-L1 therapy.

Nevertheless, deciphering the complex interactive processes between tumor and stroma cells in PAAD warrants considerable further study. Although investigations that focus on the target-specific elimination of cancerous cells or pro-tumorigenesis components of the tumor microenvironment will undoubtedly contribute to identifying specific treatment options, future research should not be limited to singular components of PAAD. Studies on multiple compartments of the tumor microenvironment that evaluate combinations of drugs that target both tumors and stroma, as well as the inhibition of cancer-promoting signaling pathways and checkpoints, will aid in developing therapeutic targets and agents that are cytotoxic to cancerous cells and activate anti-tumor immune responses. In PAAD in particular, approaches designed to overcome the current therapeutic dilemma by suppressing desmoplastic responses, overcoming immunosuppression, and inhibiting tumor-promoting signaling pathways, may provide novel therapeutics that will comtribute to enhancing the overall treatment outcomes of PAAD patients.

## Author contributions

ZC, C, S-lS, and YS contributed to the conception and design of the review. C-sC and P-wY wrote the first draft of the manuscript. YS and S-lS wrote sections of the manuscript. All authors contributed to the article and approved the submitted version. 

## Funding

This work was supported by the National Natural Science Foundation of China (81930115), Shanghai Science and Technology Commission (22YF1425800), and the Natural Science Foundation of Shanghai (21ZR1481800).

## Conflict of interest

The authors declare that the research was conducted in the absence of any commercial or financial relationships that could be construed as a potential conflict of interest.

## Publisher’s note

All claims expressed in this article are solely those of the authors and do not necessarily represent those of their affiliated organizations, or those of the publisher, the editors and the reviewers. Any product that may be evaluated in this article, or claim that may be made by its manufacturer, is not guaranteed or endorsed by the publisher.

## Abbreviations

FAP, fibroblast activation protein-α; APCE, antiplasmin-cleaving enzyme; CAFs, cancer-associated fibroblasts; PAAD, pancreatic adenocarcinoma; CPTAC, national cancer institute clinical proteomic tumor analysis consortium; PDAC, pancreatic ductal adenocarcinoma; DPP, dipeptidyl peptidase; ECM, extracellular matrix; BIBH 1, humanized version unconjugated sibrotuzumab; CAR-T, chimeric antigen receptor T; CXCL12, chemokine (C-X-C motif) ligand 12; CXCR4, chemokine (C-X-C motif) receptor 4; MDSCs, myeloid-derived suppressor cells; CCR2, CCL2 receptor; TAM, tumor-associated macrophage; HO-1, heme oxygenase-1; VEGF, vascular endothelial growth factor; TNF, tumor necrosis factor; PSCs, pancreatic stellate cells; TGFβ1, transforming growth factor-β; CTLA-4, cytotoxic T-lymphocyte-associated protein 4; PD-1, programmed cell death protein 1; PD-L1, programmed cell death protein ligand 1; CAT-T cells, chimeric antigen receptor T cells; CAP, cleavable amphiphilic peptide; BMSCs, bone marrow stromal cells; lncRNAs, long non-coding RNAs.
